# Junior Rounds: an educational initiative to improve role transitions for junior residents

**DOI:** 10.1186/s13104-017-3027-5

**Published:** 2017-12-06

**Authors:** Richard Dunbar-Yaffe, Wayne L. Gold, Peter E. Wu

**Affiliations:** 1 Division of General Internal Medicine, University of Toronto, Toronto, Canada; 2 Division of Infectious Diseases and General Internal Medicine, University of Toronto, Toronto, Canada; 3 Toronto General Hospital, 200 Elizabeth St., Eaton Building 13EN-213, Toronto, ON M5G 2C4 Canada

## Abstract

**Objective:**

At our institution, Morning Report focuses mostly on diagnostic reasoning. This makes it a challenge for first-year residents to learn to manage common on-call emergencies, such as hyperkalemia. We sought to improve their preparedness for the transitions they would encounter: from medical student to physician at the beginning of the academic year, and from junior resident to senior resident toward the end. In response to feedback, we developed the *Junior Rounds* curriculum: a weekly session focused on the approach to commonly encountered on-call emergencies and internal medicine referrals. Anonymous surveys were sent to trainees, and iterative analysis of monthly feedback led to changes to *Junior Rounds.*

**Results:**

*Junior Rounds* was implemented from August 2015 to June 2016. Thirty-nine of 92 possible respondents (44%) completed surveys in that period. Most respondents agreed that *Junior Rounds* met their educational needs, was presented at an appropriate level, and was more important to their learning than other available educational activities. Our experience demonstrates that dedicated time for level-specific learning aimed to support the transitions of junior residents can be successfully achieved. Iterative adjustment to these rounds based on feedback allowed for evolution of the curriculum to meet the changing priorities of junior learners.

**Electronic supplementary material:**

The online version of this article (10.1186/s13104-017-3027-5) contains supplementary material, which is available to authorized users.

## Introduction

Morning Report is a traditional educational format in many North American internal medicine training programs [1, 2]. It is characterized by a congregation of medical trainees and attending physicians on the internal medicine Clinical Teaching Unit (CTU) that occurs with regular frequency (usually daily), wherein cases are presented to a senior clinician in a stepwise fashion. Cases are discussed with the primary goal of enhancing knowledge [1, 3]. Other objectives include the promotion of high-quality patient care, departmental oversight on the internal medicine ward, discussion of medical error, including diagnostic reasoning errors and systems level issues, as well as informal trainee evaluation. While it may not be an intentional component of Morning Report, socialization and peer support are also important benefits of these rounds [1].

There is considerable variability in the content and format of Morning Report [1, 3–5]. Though duration and frequency are relatively constant, programs differ in who attends Morning Report (faculty, senior residents, junior residents, residents from other training programs, medical students, allied health professionals, librarians), who leads Morning Report, whether one or multiple cases are presented, whether the presentation is interactive or didactic, and whether diagnosis or management is the primary focus of discussion [3, 4]. Numerous interventions have sought to bolster the educational value of Morning Report by incorporating evidence and search techniques [6, 7], improving its interactive nature [5], and enhancing case-selection [8]. To our knowledge, no intervention has focused specifically on the needs of first-year resident trainees, who face the transitions from both medical student to junior resident and junior resident to senior resident in the same academic year. These transitions may be stressful with self-perceptions of a lack of preparedness for their roles [9].

Our institution is a 417-bed university-affiliated hospital in Canada where Morning Report occurs daily lasting 45-min. All trainees on CTU attend Morning Report. This includes medical students in their third and fourth years of training, first, second and third-year residents, and the chief medical resident (a fourth-year resident). Most Morning Report sessions are led by attending physicians. Occasionally the chief medical resident or senior subspecialty resident will lead Morning Report. Except for the faculty member facilitating Morning Report, additional faculty rarely attend Morning Report at our hospital. In contrast to published descriptions of other Morning Report programs [4], a single case is usually presented to the facilitator in a step-wise fashion by one of the trainees. The facilitator facilitates discussion to engage in Socratic teaching with the trainees. Subspecialists such as rheumatologists, nephrologists, or cardiologists occasionally lead Morning Report with cases that are preselected to match their clinical expertise. At our institution, the emphasis of discussion is on diagnostic reasoning with a minority of time spent on specifics of management.

We received informal feedback from junior (first-year) residents who regularly attended Morning Report suggesting that there was insufficient focus on the management of common medical problems and emergencies encountered on-call. Comfort with these scenarios was felt to be of paramount importance to junior trainees who had just transitioned from medical student to resident, and who would transition from junior to senior resident by the next academic year. This feedback was explored and corroborated during debriefing sessions of the first two blocks of the 2015–2016 academic year wherein the chief medical resident (RDY) solicited ideas on how to improve the CTU educational experience. The suggestion for dedicated teaching time for junior residents originated during these sessions. To address this gap, we developed a pragmatic, level-specific educational curriculum to prepare learners for their new responsibilities.

## Main text

### Methods

Between August 2015 and June 2016, the chief medical resident and a senior subspecialty resident facilitated a once-weekly, 45-min educational session restricted to junior residents (*Junior Rounds*) at a university-affiliated teaching hospital in Toronto, Ontario, Canada in lieu of their attendance at the traditional multi-level Morning Report, which still occurred concurrently for other learners. Residents were from both internal medicine and non-internal medicine specialties. *Junior Rounds* attendance ranged from 3 to 8 residents.

Teaching of practical approaches to ward-based emergencies (e.g. hyperkalemia, seizures) and common Emergency Department referrals (e.g. upper gastrointestinal bleeding, decompensated heart failure) formed the basis of the curriculum. Whenever possible, guideline and evidence-based recommendations were taught to trainees. Monthly anonymous surveys were conducted to seek real-time feedback. *Junior Rounds* were iteratively adjusted based on analysis of feedback. The decision to deliver *Junior Rounds* once-weekly in lieu of Morning Report was pragmatic to make use of already protected teaching time. As this educational need was identified at the beginning of the academic year, we favoured an approach of early curricular implementation followed by adjustments based on feedback.

**Fig. 1:**
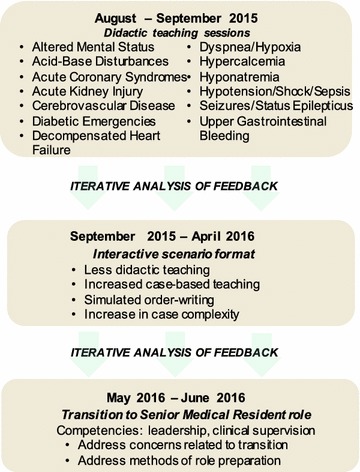
*Junior Rounds* began in August 2015 with didactic teaching related to the management of common internal medicine problems. Feedback from this initial period led to the more learner-centred, interactive, scenario-based teaching format about the same topics for the remainder of the academic year. Finally, as residents’ priorities shifted related to their upcoming transition to senior resident, the focus of the curriculum moved from acute management of medical issues to preparing for the senior resident role

*Junior Rounds* was initially trialed with a didactic teaching approach and subsequently adjusted to an interactive cased-based format in response to feedback from junior residents that the application of this knowledge to clinical care was more important to their development (Fig. 1). This format was loosely modeled on the Objective Structured Clinical Examination component of the Royal College of Physicians and Surgeons of Canada certification examination in Internal Medicine where scenarios are used to assess clinical management [10]. Residents were presented with medical scenarios (e.g. hyponatremia) and asked to outline their investigative and management steps. Simulation of order writing was employed, requiring residents to commit to management plans in concrete terms, which helped to identify knowledge gaps. Summary handouts were created as a supplement for on-call reference.

Self-identified learning needs evolved over the course of the academic year as junior residents prepared for their next transition to senior resident. Therefore, the initial focus on medical expertise shifted to include teaching on communication, leadership and clinical supervision, and methods for “surviving call” as a senior internal medicine resident. The audience for *Junior Rounds* was also narrowed to include only trainees from internal medicine for the remainder of the academic year (May and June 2016).

Surveys were sent electronically by email to junior resident trainees on a monthly basis. Data were collected anonymously (see Additional file 1: Appendix S1). Surveys contained questions based on a 5-point Likert scale. Qualitative feedback and comments could also be provided. The Research Ethics Board of the University Health Network waived the need for institutional review.

### Results

For the academic year from August 2015 to June 30, 2016, 39 out of a possible 92 respondents completed the survey (Table 1) for a response rate of 42%. Junior residents were eligible to complete the survey on more than one occasion if they rotated on the CTU for more than one rotation. This was decided in an attempt to capture evolving themes and feedback on the changes that were made. Twenty-two of 39 respondents were internal medicine trainees (59%) and the remainder was from other residency programs rotating through the CTU (e.g. Obstetrics and Gynecology, Medical Genetics, etc.).

**Table 1:** Survey questions and responses by first-year residents regarding *Junior Rounds*

Question	Responses—No. (%)				
I am currently a PGY-1 in	Internal Medicine Training Program other than internal medicine	26 (59%) 18 (41%)			
The topics presented at Junior Rounds reflected my learning needs	Strongly agree—34 (77)	Somewhat agree—9 (20)	Neutral—1 (2)	Somewhat disagree—0	Strongly disagree—0
Junior Rounds were presented at an appropriate level for my learning needs	Strongly agree—36 (80)	Somewhat agree—7 (16)	Neutral—2 (4)	Somewhat disagree—0	Strongly disagree—0
I have applied the knowledge learned at Junior Rounds to the care of my patients on this rotation	Strongly agree—31 (69)	Somewhat agree—10 (22)	Neutral—4 (9)	Somewhat disagree—0	Strongly disagree—0
I have referred to the “handouts” from Junior Rounds following the session(s)	Strongly agree—16 (36)	Somewhat agree—12 (27)	Neutral—13 (30)	Somewhat disagree—3 (7)	Strongly disagree—0
Junior Rounds were an important component to my learning on the Clinical Teaching Unit	Strongly agree—26 (58)	Somewhat agree—17 (38)	Neutral—1 (2)	Somewhat disagree—1 (2)	Strongly disagree—0
In relation to other scheduled educational rounds on the Clinical Teaching Unit, Junior Rounds have been	More important to my learning—27 (60)	As important to my learning—18 (40)	Less important to my learning—0		

Numerical values following each response reflect the number with the percent of total in brackets. All survey responses received during the 2015—2016 academic year are included in this table

The vast majority of respondents (95%) agreed or strongly agreed that *Junior Rounds* reflected their level-specific learning goals. Almost all respondents (95%) also agreed that *Junior Rounds* were presented at an appropriate level for their learning needs. When asked about the learning value of *Junior Rounds* in relation to other learning activities on the CTU (Morning Report, Noon Rounds—dedicated lectures, Attending Rounds, etc.), 40% of respondents agreed that they were equally important, while 60% of respondents agreed that they were more important to their learning needs. Most respondents (92%) either somewhat agreed or strongly agreed that they had used the information learned in *Junior Rounds* while on-call to help with clinical decision-making. Slightly more than half of respondents (58%) either somewhat agreed or strongly agreed that they had referenced the provided handouts.

### Discussion

The transition from medical student to junior resident may be stressful. Trainees often have self-perceptions of inadequate preparation for their new roles [9]. While Morning Report, a common teaching and learning format, serves several important educational goals, our learners did not feel it sufficiently addressed their preparedness for their new clinical roles.

We present a report of an educational innovation called *Junior Rounds* which was perceived to be helpful to junior residents rotating through the CTU at our hospital. The content provided in *Junior Rounds* focused most heavily on management of common medical scenarios encountered in the emergency department and on the medical wards. This contrasted significantly with Morning Report at our institution, in which clinical reasoning, differential diagnosis and step-wise presentation of a clinical case is standard. Our intervention was new and depended heavily on the input from junior residents in its initial design and subsequent improvements. Results of our monthly surveys were reviewed and were used to make real-time changes based on learner feedback.

*Junior Rounds* was perceived by trainees to reflect their level-specific learning needs and helped them perform their daily clinical activities. Many trainees felt that these rounds were more important and useful than traditional Morning Report (which they still attended the remaining days of the week). While most trainees (95%) agreed that the content of *Junior Rounds* was important, post-session reference to the handouts was modest, with only about half of the trainees reporting regular referral to them. Part of this may have been related to availability, as only paper copies were initially distributed. Subsequently, handouts were compiled and made available electronically for easy access online and on handheld devices. Substantial time and effort (approximately 20 h) was invested into the preparation of the handouts. It will be re-evaluated to determine whether electronic distribution has proved more useful to trainees.

The success of this educational intervention may largely reflect the learner-directed design and delivery [11] that allowed residents to assume control of their own learning needs [12]. Demonstration of the program’s responsiveness to feedback also contributed to the success of this intervention [13].

From a delivery perspective, *Junior Rounds* proved feasible as it was usually led by either the chief medical resident, a senior subspecialty trainee, or an attending physician, who were committed to the success of this educational endeavor. That said, one additional facilitator per week was required as traditional Morning Report ran concurrently for the other levels of learners.

While we did not specifically ask about any negative impact of missing Morning Report once per week, we believe that this was not the cases as 60% of respondents felt that *Junior Rounds* were more important to their learning needs than other educational rounds. Furthermore, the smaller number of participants also allowed for camaraderie and socialization, an aspect of Morning Report that is frequently highlighted [1].

There may be unintended consequences of *Junior Rounds*. Other learners who attend Morning Report (senior residents, medical students) were not specifically asked to provide feedback regarding the absence of their colleagues. Attending physicians leading Morning Report were sometimes disappointed with the lower attendance at Morning Report on mornings when *Junior Rounds* ran concurrently and occasionally, when invited faculty from subspecialties services led Morning Report, *Junior Rounds* were cancelled to facilitate fuller attendance.

### Conclusion

While Morning Report remains a valued educational event in most internal medicine training programs, it is challenged with the task of satisfying the educational needs of multiple levels of learners. This single-centre educational initiative for junior residents demonstrates that dedicated time for level-specific learning aimed to support the transitions from medical student to junior resident and from junior to senior resident can be successfully achieved. *Junior Rounds* provided timely teaching on the management of common internal medicine issues, as well as a safe space for learners to make mistakes and ask questions. Finally, iterative adjustment based on monthly feedback allowed for dynamic evolution of the curriculum to meet the changing needs of junior learners as they progressed through their first year of residency.

### Limitations

There are several limitations to our intervention. *Junior Rounds* was implemented at one of the six university-affiliated teaching hospitals with its evaluation based on 44 resident responses. The relatively small sample size consisting of both internal medicine and non-internal medicine residents means our findings may not be generalizable to other training models, including those comprised exclusively or predominantly of internal medicine residents. Notably, we received feedback from 22 internal medicine residents, which is of comparable size to many other training programs. Also, the content of *Junior Rounds* may be presented in other formats at other hospitals, including traditional Morning Report where management strategies may be highlighted. While we could have chosen to increase the time spent on management strategies at our own Morning Report, we also believed that it was important for the junior learners to have safe educational space, where they were not being compared to medical students or senior residents and might feel greater freedom to express uncertainty. If other institutions choose to implement a similar educational format, this would be important to evaluate. Finally, while perceptions of the value of *Junior Rounds* were subjective, next phases should include an assessment of knowledge acquisition and application, which might include scenario-based assessments.

## Additional file

**Additional file 1: Appendix S1.** *Junior Rounds* 2015–2016 survey questions.

## Abbreviation

CTU: Clinical Teaching Unit
