# Supplementary material for: Junior Rounds: an educational initiative to improve role transitions for junior residents
Source: BMC Res Notes. 2017 Dec 6;10:694. doi: 10.1186/s13104-017-3027-5 (PMC5717831; doi:10.1186/s13104-017-3027-5)
Supplement: Supplementary file 1 — Additional file 1: Appendix S1. Junior Rounds 2015–2016 survey questions. [file 13104_2017_3027_MOESM1_ESM.docx]

Appendix S1 – *Junior Rounds* 2015-2016 Survey Questions

**I am currently a PGY-1 in:**

Internal Medicine

Other Residency Program

*Using the provided scale, please rank your level of agreement with the following statements:*

**The topics presented at Junior Rounds reflected my learning needs.**

| Strongly Agree | Agree | Neutral | Disagree | Strongly Disagree |
| --- | --- | --- | --- | --- |

**Junior Rounds were presented at an appropriate level for my learning needs.**

| Strongly Agree | Agree | Neutral | Disagree | Strongly Disagree |
| --- | --- | --- | --- | --- |

**I have applied the knowledge learned at Junior Rounds to the care of my patients on this rotation.**

| Strongly Agree | Agree | Neutral | Disagree | Strongly Disagree |
| --- | --- | --- | --- | --- |

**I have referred to the "handouts" from Junior Rounds following the session(s).**

| Strongly Agree | Agree | Neutral | Disagree | Strongly Disagree |
| --- | --- | --- | --- | --- |

**Junior Rounds were an important component to my learning on the Clinical Teaching Unit.**

| Strongly Agree | Agree | Neutral | Disagree | Strongly Disagree |
| --- | --- | --- | --- | --- |

**In relation to other scheduled educational rounds on the Clinical Teaching Unit, Junior Rounds have been:**

| More important to my learning | As important to my learning | Less important to my learning |
| --- | --- | --- |

**Please enter any other comments you may have about junior rounds in the box below:**

|  |
| --- |
